# Lentivirus-mediated LIGHT overexpression inhibits human colorectal carcinoma cell growth *in vitro* and *in vivo*

**DOI:** 10.3892/ol.2013.1505

**Published:** 2013-08-01

**Authors:** HAIBO WANG, ZHUANG YU, SHIHAI LIU, XIANGPING LIU, AIHUA SUI, RUYONG YAO, ZHENG LUO, CHUANZHI LI

**Affiliations:** 1 Center of Diagnosis and Treatment of Breast Disease, The Affiliated Hospital of Medical College, Qingdao University, Qingdao, Shandong 266003, P.R. China; 2 Department of Oncology, The Affiliated Hospital of Medical College, Qingdao University, Qingdao, Shandong 266003, P.R. China; 3 Central Laboratory of Molecular Biology, The Affiliated Hospital of Medical College, Qingdao University, Qingdao, Shandong 266003, P.R. China; 4 Department of General Surgery, The First People’s Hospital of Jining, Jining, Shandong 272011, P.R. China; 5 Department of General Surgery, Central Hospital of Jinan, Jinan, Shandong 250013, P.R. China

**Keywords:** LIGHT/TNFSF14, lentiviral vector, colorectal carcinoma cell, xenografted tumor

## Abstract

Human LIGHT (lymphotoxin-related inducible ligand that competes for glycoprotein D binding to herpesvirus entry mediator on T cells) is the 14th member of the tumor necrosis factor (TNF) superfamily and is therefore also known as TNFSF14. LIGHT has been proven to be a multifunctional molecule affecting cell proliferation, differentiation and a number of other biological processes, in particular, cell growth inhibition. However, the expression and molecular mechanisms of the LIGHT gene in human colorectal carcinoma cells remain largely unclear. In the present study, the LIGHT gene was overexpressed using a lentiviral expression vector in HCT116 human colorectal carcinoma cells *in vitro* and *in vivo,* in order to explore the mechanism by which the LIGHT gene inhibits cell growth and suppresses tumor formation. The results showed that the recombinant lentivirus with LIGHT overexpression inhibited the proliferative capacity of the HCT116 cells and significantly decreased the xenografted tumor volumes in nude mice. Furthermore, LIGHT treatment effectively initiated increased caspase-3 and decreased Bcl-2 activities in the HCT116 cells. This study provides a basis for the improved understanding of the role and molecular mechanisms of the LIGHT gene in human colorectal carcinoma cells and may facilitate further functional studies of LIGHT.

## Introduction

Human LIGHT (lymphotoxin-related inducible ligand that competes for glycoprotein D binding to herpesvirus entry mediator on T cells) is the 14th member of tumor necrosis factor (TNF) superfamily and is therefore also referred to as TNFSF14 (1). LIGHT is able to bind the lymphotoxin-β receptor (LTβR), which is expressed on numerous types of epithelial cancer, and the herpes virus entry mediator (HVEM), a receptor expressed by T lymphocytes; therefore, LIGHT is additionally known as HVEM-L (herpesvirus entry mediator-ligand) (2). LIGHT is a multifunctional molecule affecting cell proliferation, differentiation and a number of other biological processes (3). LIGHT costimulates T-cell amplification effects and enhances the cell immune reaction to tumors (4), acting as the most effective tumor immunotherapy factor (5,6). However, the mechanism by which LIGHT affects cancer cells is poorly understood.

A previous study by our group showed that LIGHT promotes HepG2 hepatic carcinoma cell apoptosis through regulating the expression of Bcl-2 and caspase-8 (7). Furthermore, LIGHT was not detected in the HCT116 colorectal cancer cell line and the overexpression of LIGHT transfected into HCT116 cells by plasmid vector inhibited cell growth. Due to the low transfection efficiency of plasmids, a stable cell line with LIGHT overexpression may facilitate further functional studies of LIGHT.

In the present study, LIGHT was overexpressed using a lentiviral expression vector in the HCT116 colorectal cancer cell line and its effects on cell biology were investigated, thus providing a basis for further study of LIGHT functions.

## Materials and methods

### Cell line and culture

The HCT116 human colon cancer cell line was purchased from China Centre for Type Culture Collection (Shanghai, China). The cells were cultured at 37°C in McCoy’s 5α (modified) medium (Sigma, St. Louis, MO, USA), supplemented with 10% fetal bovine serum (FBS; Hyclone, Waltham, MA, USA) in a humidified atmosphere of 5% CO_2_. The cells were detached using 0.25% trypsin and 0.02% ethylenediaminetetraacetic acid (EDTA).

### Animals

Athymic nude male BALBC/c mice, weighing 17–19 g (4–5 weeks old), were purchased from the Institute of Laboratory Animal science, Chinese Academy of Medical Science (Beijing, China). The mice were maintained in specific pathogen-free, temperature-controlled isolation conditions and fed with sterilized food and autoclaved water according to the experimental animal guidelines. The use of animals in the present study complies with the Guide for the Care and Use of Laboratory Animals. All animal studies were approved by the Animal Research and Ethical Committee of Qingdao Medical College (Shandong, China).

### Construction of recombinant lentivirus vector encoding the LIGHT gene

Primers for whole length cDNA of human LIGHT were designed and synthesized with sequences as follows: Sense, 5′-CGCGGATCCATGGAGGAGAGTGTCG-3′ and antisense, 5′-CTCGTCGACTCACACCATGAAAGCCCC-3′. Briefly, the LIGHT gene was amplified by HotStar Taq DNA polymerase (Qiagen, Hilden, Germany) using the pAAV-LIGHT plasmid as a template and then the LIGHT gene and pLenti vector were cut by *Sal*I and *Bam*HI enzymes. Following recycling electrophoresis, the LIGHT gene was subcloned into the pLenti plasmid and the recombined plasmid, pLenti-LIGHT, was identified by incision with the *Sal*I and *Bam*HI enzymes and sequencing. Primer synthesis and DNA sequencing were performed by Shanghai Shangon Co. Ltd. (Shanghai, China). The viral particles were generated by the cotransfection of 293T cells (ATCC) via a calcium phosphate-mediated transfection method with pLenti-LIGHT or pLenti-GFP and three packaging vectors. Three days after transfection, the cell culture supernatants were harvested (1,600 × g for 5 min), filtered through 0.45-μm pore size filters and concentrated 100-fold by ultracentrifugation at 7,000 × g for 16 h. The viral particles were stored in small aliquots at −80°C. The virus titer was determined by calculating the percentage of green fluorescent protein (GFP)-positive cells, as observed by fluorescence microscopy.

### Transfection and selection of stable HCT116 cell lines (HCT116/LIGHT)

For the transfection of the tumor cell lines, lentiviral vectors harboring LIGHT were constructed and the HCT116 cells were infected. Briefly, the HCT116 cells were cultured in McCoy’s 5α medium containing 10% FBS and when they reached the exponential growth phase, 1.0×10^5^ cells per well were plated in 24 plates. Next, 300 μl complete culture medium, containing recombinant lentiviruses, control lentiviruses or McCoy’s 5α medium (all containing 6 μg/ml polybrene; Sigma) was added into the plates when the cells reached 50–60% confluence. Two days later, the virus-containing medium was replaced with fresh complete medium. The expression level of GFP was observed under a microscope after 3 days. Medium containing blasticidin (6 μg/ml; Merck KGaA, Darmstadt, Germany) was added every 3 or 4 days to screen the stable infected cell lines (HCT116/LIGHT or HCT116/GFP, with clear clone formation) until the uninfected cells were almost completely removed.

### Determination of the optimal multiplicity of infection (MOI)

To assess the efficiency of lentiviral transduction in the human HCT116 cells, the cells were infected with pLenti-GFP at various MOIs for 24 h. The supernatant was then changed to fresh complete medium every other day. After 72 h, GFP-expressing cells were detected by fluorescence microscopy (Olympus; Tokyo, Japan).

### Semi-quantitative reverse transcription-PCR analysis

The total RNA of HCT116/LIGHT, HCT116/GFP or the control cells was extracted using RNAiso reagent (Takara, Japan), and then converted into cDNA using a PrimeScript™ RT reagent kit (Takara), according to the manufacturer’s instructions. The specific oligonucleotide primers of the LIGHT (PCR product 128 bp) and GAPDH (PCR product 151 bp) genes were as follows: Sense, 5′-GTACGGCCCTCAGTGTTTGTG-3′ and antisense, 5′-CCCATCAGCAACAGCAAGAGA-3′; and sense, 5′-CTTAGCACCCCTGGCCAAG-3′ and antisense, 5′-GATGTTCTGGAGAGCCCCG-3′, respectively. The reaction conditions of pre-denaturation were 95°C for 3 min, 95°C for 30 sec, 60°C for 30 sec and 72°C for 30 sec, with 22 cycles for GAPDH and 29 cycles for LIGHT (the cycles were based on PCR kinetics) and a total reaction volume of 20 μl. Each PCR was repeated three times. The products were subsequently analyzed using 2% agarose gel electrophoresis. The semiquantitative analysis of LIGHT and GAPDH mRNA levels was measured using the Syngene Gel Imaging System and analysis software (Syngene Co., Cambridge, UK).

### Expression of LIGHT protein by ELISA

Cell supernatants were collected after 48 h and the expression of LIGHT protein was detected by ELISA according to the manufacturer’s instructions (R&D, Minneapolis, MN, USA). The kit was capable of detecting LIGHT with a minimal detectable dose as low as 10 pg/ml. The primary wavelength was 450 nm (optionally 620 nm as the reference wavelength). All the tests were repeated three times with three wells per group.

### Western blot analysis

The HCT116 cells were plated onto type I collagen-coated 25-cm^2^ flasks, then treated with Lenti-GFP or Lenti-LIGHT for 48 h in basal medium containing 10% FBS. The HCT116/LIGHT, HCT116/GFP or control cells were harvested with a cell scraper, and stored at −80°C until protein extraction. The pellets were resuspended with a lysis buffer [50 mM Tris-HCl (pH 8.0), 1 mM EDTA, 150 mM NaCl, 1% Nonidet P-40, 100 μm 4-amidinophenylmethanesulfonyl fluoride, 1 μg/ml aprotinin, 5 μg/ml leupeptin, 1 μg/ml pepstatin A and 50 μg/ml antipain] and then mixed well at 4°C. Following centrifugation, the protein concentration of each supernatant was determined using the Bio-Rad Protein Assay (Bio-Rad, Hercules, CA, USA). Samples were subjected to 10% SDS-PAGE and subsequently transferred to polyvinylidene difluoride membranes (Millipore, Bedford, MA, USA) in a transfer buffer. These membranes were blocked in BlockAce (Dainippon Seiyaku, Japan) overnight at 4°C. Rabbit anti-caspase-3, -Bcl-2 or -GAPDH antibodies (Abcam, Cambridge, MA, USA) were used as primary antibodies at a 1:1,000 dilution in 10% BlockAce for 30 min. The samples were then washed in phosphate-buffered saline containing 0.05% Tween-20 (Bio-Rad). Goat anti-rabbit IgG was used as secondary antibody at a 1:5,000 dilution for 30 min. An enhanced chemiluminescence (ECL) detection system (Pierce Biotechnology Inc., Rockford, IL, USA) was then used to visualize immunoreactive protein complexes. An autoradiograph was obtained and the protein levels were measured using a FluorS scanner and Quantity One software for analysis (Bio-Rad).

### Analysis of cell proliferation (8)

The cells (1.0×10^3^/well) were plated and treated in 96-well plates (three wells per group, total 5 plates) for 24, 48, 72, 96 and 120 h, respectively. At the indicated times, the medium was removed and fresh medium containing 3-(4,5-dimethylthiazol-2-yl)-2,5-diphenyltetrazolium bromide (MTT, 0.5 mg/ml; Sigma) was added to each well. The cells were incubated at 37°C for 4 h and then the medium was removed and 150 μl solubilization solution (DMSO) was added and mixed thoroughly. Absorbance from the plates was read on a Safire II spectrometer reader (Tecan, Männedorf, Swiss) at 490 nm (8).

### Subcutaneous human colorectal cancer cell xenograft growth and oncogenicity

Balb/c nude mice were maintained in specific pathogen-free, temperature-controlled isolation conditions, and fed with sterilized food and autoclaved water. Subsequent to being washed with serum-free McCoy’s 5α medium, the cells were collected and 200 μl cell solution (1.0×10^7^ cells in 200 μl PBS, with a viability of >95%) was injected subcutaneous1y into the right-side of the backs of the mice. The sizes of the transplanted tumor xenograft were measured periodically until the long diameter of tumor xenograft was >1 cm. The tumor xenografts were then dissected and measured with a vernier caliper. The tumor volumes were calculated using the following formula: Tumor volume = long diameter × short diameter^2^ / 2).

### Statistical analysis

All data were shown as the mean ± SD unless otherwise mentioned. Statistical analyses were performed using SPSS 11.5 (SPSS Inc., Chicago, IL, USA). Differences were assessed between the two groups using a t-test. P<0.05 was considered to indicate a statistically significant difference.

## Results

### Identification of recombinant lentiviral vectors and GFP visualization

**Figure 1:**
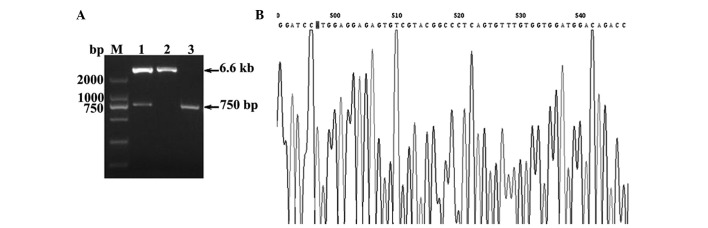
(A) Identification of recombined lentiviral vector, pLenti-LIGHT. M, DL2000 DNA marker; Lane 1, plasmid pLenti-LIGHT digested with *Sal*I and *Bam*HI; lane 2, plasmid pLenti digested with *Sal*I and *Bam*HI; lane 3, PCR products of LIGHT; LIGHT, lymphotoxin-related inducible ligand that competes for glycoprotein D binding to herpesvirus entry mediator on T cells. (B) Sequencing histograms of pLenti-LIGHT.

The 1.5% agarose gel electrophoresis showed a LIGHT gene product, recombinant plasmid and pLenti plasmid (plasmids were incised by *Sal*I and *Bam*HI), as shown in Fig. 1. The PCR product of the LIGHT gene was ~750 bp and the recombinant plasmid exhibited 6.6 kb and 750 bp products after enzyme incision. The sequence of the LIGHT gene was demonstrated to be the same compared with the sequence from GenBank (Fig. 1).

**Figure 2:**
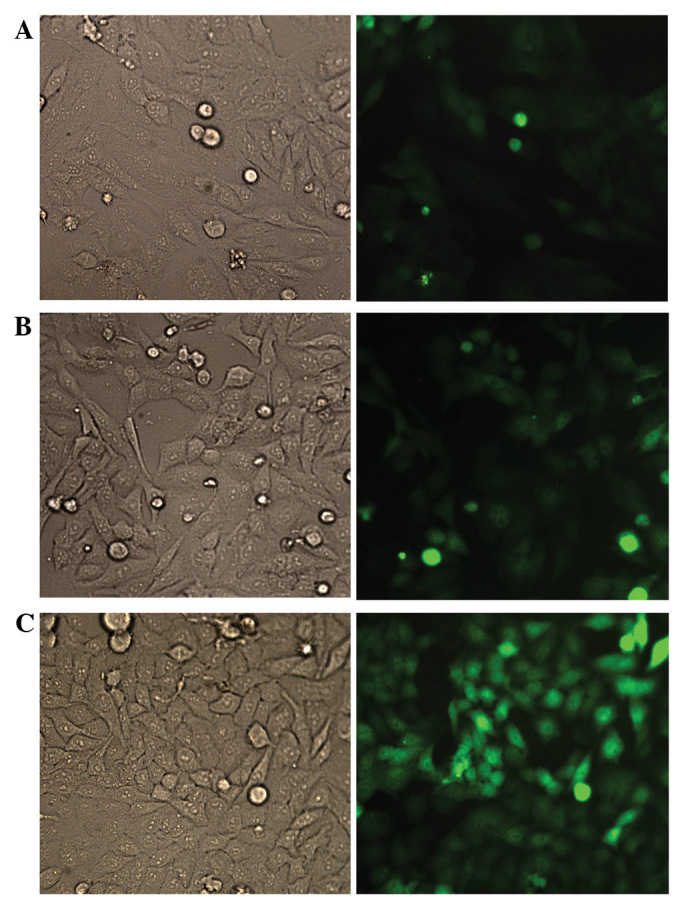
Green fluorescent protein (GFP) visualization of HCT116 cells transduced with Lenti-GFP. HCT116 cells were transduced with Lenti-GFP at different multiplicities of infection (MOI). At 72 h post-transduction, GFP-expressing cells were imaged by fluorescence microscopy (magnification, ×200; left column, bright field; right column, fluorescence vision). (A) MOI, 2. (B) MOI, 5. (C) MOI, 8.

The HCT116 cells exhibited a high lentiviral transduction efficiency at a MOI of 5 at 72 h. The visualized GFP results showed that the number of GFP-expressing cells increased in an MOI-dependent manner (Fig. 2). As an MOI of 5 was shown to be optimal for cell infection, an MOI of 5 was selected for subsequent studies. In total, ~90–95% of cells in the HCT116/LIGHT and HCT116/GFP cell lines infected with lentivirus expressed significant fluorescent signals.

### mRNA levels of LIGHT in the cells stably transfected with LIGHT

**Figure 3:**
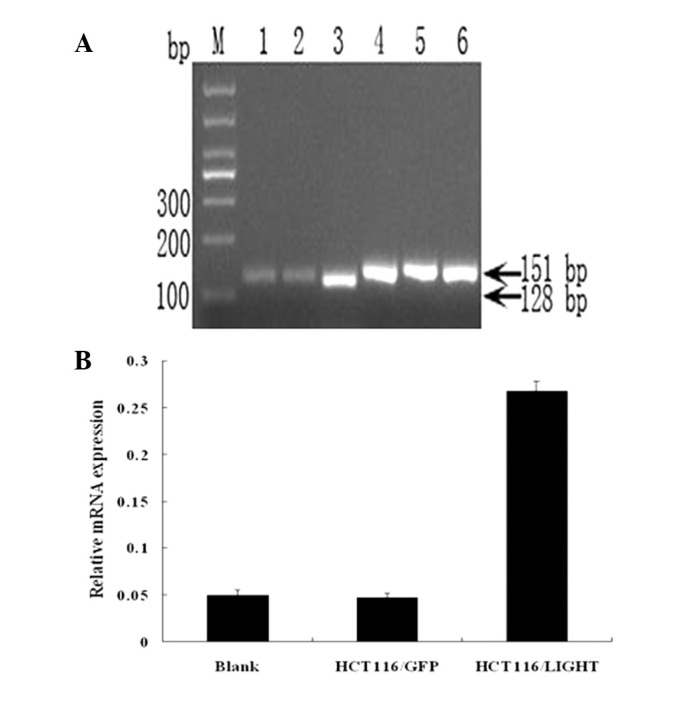
Effects of overexpressing LIGHT on the mRNA level of LIGHT. (A) Electrophoresis and semiquantitative analysis of PCR products. The mRNA level of LIGHT was higher in the HCT116/LIGHT cells compared with the HCT116/GFP and HCT116 cells. Lanes 1 and 4, HCT116; lanes 2 and 5, HCT116/GFP; lanes 3 and 6, HCT116/LIGHT; M, DL1000 DNA marker. (B) Statistical analysis of the mRNA level of LIGHT relative to that of GAPDH. LIGHT, lymphotoxin-related inducible ligand that competes for glycoprotein D binding to herpesvirus entry mediator on T cells.

The number of PCR cycles required for LIGHT and GAPDH was 29 and 22, respectively, as determined by the amplification dynamic experiments (data not shown). The lengths of the PCR products were 128 bp (LIGHT) and 151 bp (GAPDH), respectively. The PCR product electrophoresis and semiquantitative analysis showed that the relative mRNA level of LIGHT in the HCT116/LIGHT cell line was significantly higher than that of the blank control (~5.42 times more than that of the blank control) and HCT116/GFP cells (Fig. 3).

### Overexpression of LIGHT protein

The A_450_ values in the HCT116/GFP and blank control groups showed no difference with the use of McCoy’s 5α complete medium, which indicated that endogenous LIGHT in the HCT116 cells was not expressed or was weakly expressed (<10 pg/ml). The protein levels of LIGHT in the HCT116/LIGHT cells were significantly increased compared with the HCT116/GFP and blank control groups (3.16±0.15 vs. 0 ng/ml).

### LIGHT activates caspase-3 and inhibits Bcl-2 activation in HCT116 cells

**Figure 4:**
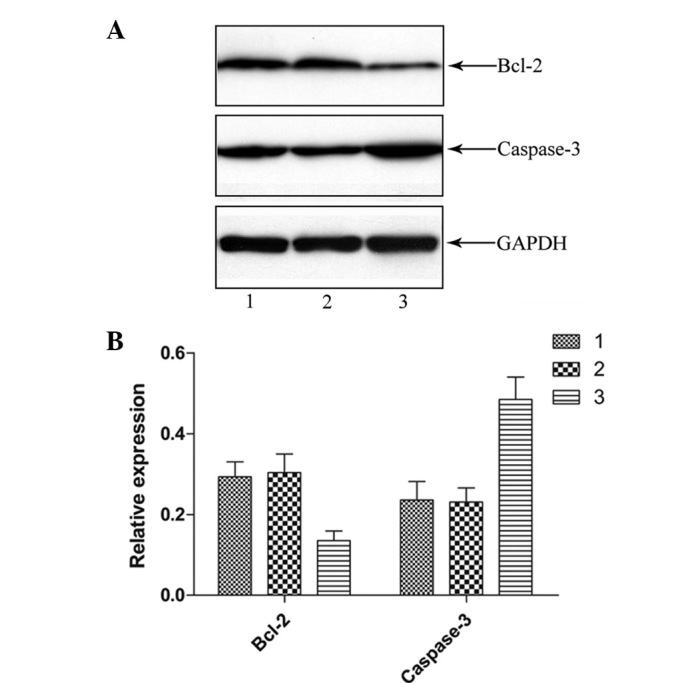
LIGHT transfection reduced caspase-3 and Bcl-2 protein levels in HCT116 cells. (A) Western blot showing caspase-3, Bcl-2 and GAPDH (loading control) staining for untreated HCT116 (lane 1), Lenti-GFP (lane 2) or Lenti-LIGHT-treated HCT116 cells (lane 3). Band signals from caspase-3 and Bcl-2 were normalized to those from GAPDH. (B) Relative expression of caspase-3 and Bcl-2 in the three groups. Lenti-LIGHT-treated HCT116 cells showed higher caspase-3 and lower Bcl-2 protein expression compared with untreated and Lenti-GFP-treated HCT116 cells (P<0.05). No difference was observed between the untreated and Lenti-GFP-treated HCT116 cells (P>0.05). LIGHT, lymphotoxin-related inducible ligand that competes for glycoprotein D binding to herpesvirus entry mediator on T cells.

Several caspases play significant roles in TNF family-mediated apoptosis. To evaluate the anti-apoptotic effect of LIGHT on the HCT116 cells, a western blot analysis was performed to investigate the processing of caspase-3 using cell lysates from the HCT116 cells pretreated with or without LIGHT. At 48 h after Lenti-GFP or Lenti-LIGHT incubation, the cleavage product of caspase-3 was clearly detected, indicating that caspase-3 activation had occurred by that time (Fig. 4). Furthermore, lower levels of Bcl-2 remained as compared with the unpretreated control. There was no difference in the quantity of cleavage products or Bcl-2 observed in the reactions with Lenti-GFP and the untreated control.

### Effect of LIGHT on HCT116 cell proliferation

**Figure 5:**
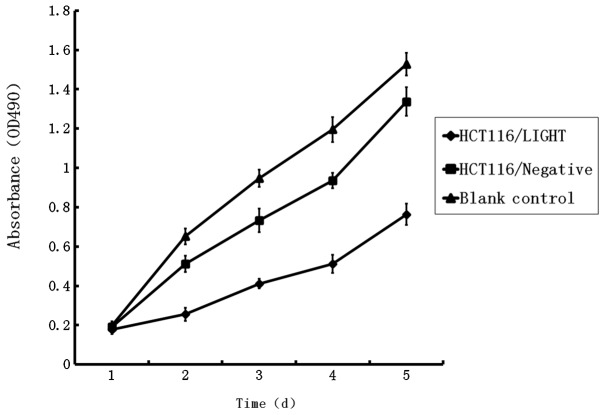
Overexpression of LIGHT suppresses HCT116/LIGHT cell proliferation. Cells were seeded at a density of 1.0×10^3^/well in 96-well plates in 100 μl DMEM. Cell proliferation was determined at 24, 48, 72, 96 and 120 h, respectively. Absorbance from the plates was read at 490 nm. Samples were performed in triplicate and data are shown as the mean ± SD. LIGHT, lymphotoxin-related inducible ligand that competes for glycoprotein D binding to herpesvirus entry mediator on T cells; DMEM, Dulbecco’s modified Eagle’s medium.

Growth curves showed a slight deceleration of cell growth in the HCT116/GFP group compared with the blank control group. There was significant growth inhibition in the HCT116/LIGHT group compared with other groups, which indicated that the expression of LIGHT caused inhibition of cell growth (Fig. 5).

### Effect of LIGHT on oncogenicity in nude mice

**Figure 6:**
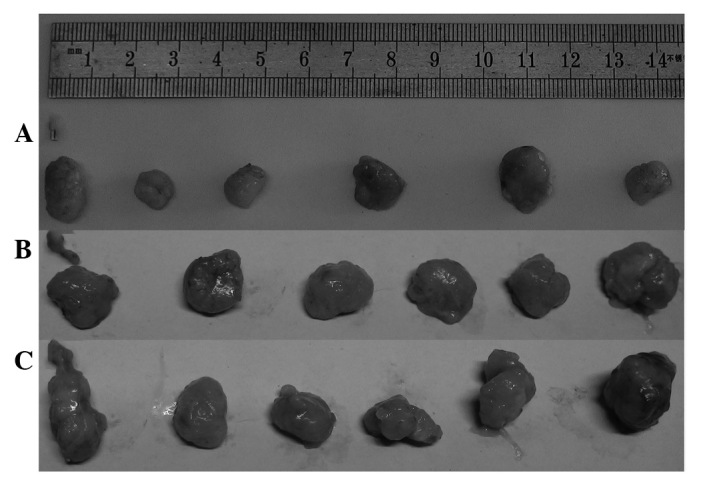
Comparison of the size of the harvested implanted tumors in nude BALBC/c mice. (A) Tumors transplanted with the HCT116/LIGHT cells. (B) Tumors transplanted with the HCT116/GFP cells. (C) Tumors transplanted with the HCT116 cells. LIGHT, lymphotoxin-related inducible ligand that competes for glycoprotein D binding to herpesvirus entry mediator on T cells.

Eight days after subcutaneous cell injection, the tumor xenografts were successfully inoculated and the volumes of the tumor xenografts became enlarged with the progression of time. The tumor volume in the HCT116/LIGHT group was smaller than in the HCT116/GFP and blank control groups (3.08 times in the blank control group and 2.80 times in the HCT116/GFP group as compared with that in the HCT116/LIGHT group; Fig. 6).

## Discussion

Colorectal cancer is a type of malignant tumor with a high clinical incidence (9). Despite surgical resection, systemic chemotherapy and radiotherapy, tumor recurrence or metastasis frequently occurs due to the incomplete elimination of tumor cells and the inadvertent impairment of normal tissue and cells. The immunotherapy of tumors may stimulate and reinforce the immune system of the body and thus control and/or kill tumor cells. Immune responses markedly affect whether an infection is cleared or will persist to pose a risk for the development of cancer. The cytokine therapy of tumors may modulate or reinforce one and/or multiple immune cell functions after cytokine injection (10,11). Cancer immunotherapies employing the tumor necrosis factor superfamily (TNFSF) molecules exhibit antitumor effects through two predominant mechanisms, the direct killing of tumor cells and indirect killing by activating antitumor immunity. The former mechanism is limited to tumors that express the appropriate tumor necrosis factor receptor superfamily (TNFRSF) molecules, while the latter works irrespective of tumor type, so it may have broad applicability as a cancer therapy (12).

LIGHT is a lymphotoxin analogue of glycoprotein D binding to HVEM on T cells, which may be expressed on activated T cells and premature dendritic cells. LIGHT interacts with two distinct cell-membrane receptors, HVEM and LTβR, and one decoy receptor, TR6/DcR3 (1,13,14). LIGHT may costimulate T cells and induce cell apoptosis (15), and has three different receptors with various biological functions. A number of studies have confirmed that the effect of the inhibition of LIGHT on tumor cell growth and the induction of apoptosis is correlated with the expression of receptors on tumor cells. LIGHT may inhibit the growth of MDA-MB-231 breast cancer cells, HT-29 colorectal cancer cells and A375 melanoma cells through its secretion and solubility pattern (6,16). LIGHT may also induce the apoptosis of tumor cells expressing LIGHT receptors (6) and synergistically induce tumor cell apoptosis with IFN-γ (7,17). Furthermore, LIGHT may induce the expression of Mig and IP-10, chemotactic factors in antitumor angiogenesis, inhibit tumor angiogenesis and act with natural killer (NK) cells (18) and accelerate antitumor T-cell immunity, which may result in delayed growth or the spontaneous regression of tumors (4), all indicating that LIGHT may be an significant antitumor factor.

The ideal viral vector should provide efficient gene transfers, stable long-term gene expression and good biological safety. The lentivirus systems used in the present study are the third generation of lentiviral vectors and HIV-based expression vectors, which offer unique versatility and robustness as vehicles for gene delivery (19). The lentivirus vectors offer significant advantages over retroviral vectors in the process of gene delivery to target cells (20,21), they are genetically altered from wild-type HIV so as to increase their biosafety and they may transduce a wide range of cell types and integrate into the host genome in dividing and post-mitotic cells, resulting in long-term expression of the transgene *in vitro* and *in vivo*(22).

In the present study, a lentivirus coexpressing GFP and LIGHT genes was first constructed. GFP was used as a reporter gene to monitor the expression of LIGHT. LIGHT was overexpressed in the HCT116 colorectal cancer cell line using pLenti-LIGHT and then the stable and overexpressed LIGHT mRNA and protein cell lines (HCT116/LIGHT) were screened. The results indicated that the mRNA and protein levels of LIGHT in the HCT116/LIGHT cells were higher than the levels in the HCT116 cells, while the overexpression of LIGHT clearly inhibited the growth of the HCT116 cells and the oncogenicity of the cells in the nude mice. Furthermore, the present study demonstrated that LIGHT may inhibit the cellular proliferative capacity of the HCT116 cells via the upregulation of caspase-3 and the downregulation of Bcl-2.

The effective immunotherapy of tumors not only inhibits or eliminates the local tumor burden, it also induces certain types of protective immunity in the body and thus may prevent further recurrence of tumor cells. Therefore, LIGHT may serve as a promising tumor immunotherapy factor and its mechanism of action consequently requires further investigation.

## References

[1] Misawa K, Nosaka T, Kojima T, Hirai M, Kitamura T (2000). Molecular cloning and characterization of a mouse homolog of human TNFSF14, a member of the TNF superfamily. Cytogenet Cell Genet.

[2] Wang JM, Deng X, Gong W, Su S (1998). Chemokines and their role in tumor growth and metastasis. J Immunol Methods.

[3] Mauri DN, Ebner R, Montgomery RI, Kochel KD, Cheung TC, Yu GL, Ruben S, Murphy M, Eisenberg RJ, Cohen GH, Spear PG, Ware CF (1998). LIGHT, a new member of the TNF superfamily, and lymphotoxin alpha are ligands for herpesvirus entry mediator. Immunity.

[4] Tamada K, Shimozaki K, Chapoval AI, Zhu G, Sica G, Files D, Boone T, Hsu H, Fu YX, Nagata S, Ni J, Chen L (2000). Modulation of T-cell-mediated immunity in tumor and graft-versushost disease models through the LIGHT co-stimulatory pathway. Nat Med.

[5] Harrop JA, McDonnell PC, Brigham BM, Lyn SD, Minton J, Tan KB, Dede K, Spampanato J, Silverman C, Hensley P, Diprinzio R, Emery JG, Deen K, Eichman C, Chabot FM, Truneh A, Young PR (1998). Herpesvirus entry mediator ligand(HVEM-L), a novel ligand for HVEM/TR2, stimulates proliferation of T cells and inhibits HT29 cell growth. J Biol Chem.

[6] Zhai Y, Guo R, Hsu TL, Yu GL, Ni J, Kwon BS, Jiang GW, Lu J, Tan J, Ugustus M, Carter K, Rojas L, Zhu F, Lincoln C, Endress G, Xing L, Wang S, Oh KO, Gentz R, Ruben S, Lippman ME, Hsieh SL, Yang D (1998). LIGHT, a novel ligand for lymphotoxin beta receptor and TR2/HVEM induces apoptosis and suppresses in vivo tumor formation via gene transfer. J Clin Invest.

[7] Wang ZH, Wu LQ, Han B, Lu Y, Lu ZH, Liu XP, Yang K, Sui AH, Bi CY, Li JP (2008). Expression of Fas/FasL and the apoptosis of HepG2 cells transfected with LIGHT and IFN-γ. Chin J Curr Adv Gen Surg.

[8] Liu XP, Wang HB, Yang K, Sui AH, Shi Q, Qu S (2009). Inhibitory effects of adenovirus mediated tandem expression of RhoA and RhoC shRNAs in HCT116 cells. J Exp Clin Cancer Res.

[9] Jemal A, Ward E, Hao Y, Thun M (2005). Trends in the leading causes of death in the United States, 1970–2002. JAMA.

[10] Kim D, Gambhira R, Karanam B, Monie A, Hung CF, Roden R, Wu TC (2008). Generation and characterization of a preventive and therapeutic HPV DNA vaccine. Vaccine.

[11] Santin AD, Bellone S, Palmieri M, Zanolini A, Ravaggi A, Siegel ER, Roman JJ, Pecorelli S, Cannon MJ (2008). Human papillomavirus type 16 and 18 E7-pulsed dendritic cell vaccination of stage IB or IIA cervical cancer patients: a phase I escalating-dose trial. J Virol.

[12] Tamada K, Chen L (2006). Renewed interest in cancer immunotherapy with the tumor necrosis factor superfamily molecules. Cancer Immunol Immunother.

[13] Tamada K, Shimozaki K, Chapoval AI, Zhai Y, Su J, Chen SF, Hsieh SL, Nagata S, Ni J, Chen L (2000). LIGHT, a TNF-like molecule, costimulates T cell proliferation and is required for dendritic cell-mediated allogeneic T cell response. J Immunol.

[14] Yu KY, Kwon B, Ni J, Zhai Y, Ebner R, Kwon BS (1999). A newly identified member of tumor necrosis factor receptor superfamily (TR6) suppresses LIGHT-mediated apoptosis. J Biol Chem.

[15] Zou GM, Hu WY (2005). LIGHT regulates CD86 expression on dendritic cells through NF-κB, but not JNK/AP-1 signal transduction pathway. J Cell Physiol.

[16] Rooney IA, Butrovich KD, Glass AA, Borboroglu S, Benedict CA, Whitbeck JC, Cohen GH, Eisenberg RJ, Ware CF (2000). The lymphotoxin-beta receptor is necessary and sufficient for LIGHT-mediated apoptosis of tumor cells. J Biol Chem.

[17] Chen MC, Hsu TL, Luh TY, Hsieh SL (2000). Overexpression of bcl-2 enhances LIGHT and interferon-gamma-mediated apoptosis in Hep3BT2 cells. J Biol Chem.

[18] Yu p, Lee Y, Liu W, Chin RK, Wang J, Wang Y, Schietinger A, Philip M, Schreober H, Fu YX (2004). Priming of naive T cells inside tumors leads to eradication of established tumors. Nat Immunol.

[19] Tiscornia G, Singer O, Verma IM (2006). Production and purification of lentiviral vectors. Nat Protoc.

[20] Wiznerowicz M, Trono D (2005). Harnessing HIV for therapy, basic research and biotechnology. Trends Biotechnol.

[21] Lever AM, Strappe PM, Zhao J (2004). Lentiviral vectors. J Biomed Sci.

[22] Buchschacher GL, Wong-Staal F (2000). Development of lentiviral vectors for gene therapy for human diseases. Blood.

